# Epidemiological Study of Animal Leptospirosis in New Caledonia

**DOI:** 10.1155/2013/826834

**Published:** 2013-02-28

**Authors:** Cédric Roqueplo, Olivier Cabre, Bernard Davoust, Angeli Kodjo

**Affiliations:** ^1^Groupe de Travail en Epidémiologie Animale du Service de Santée des Armées, Antenne Vétérinaire de Toulon, BCMR BP 95, 83800 Toulon Cedex 9, France; ^2^Direction Interarmées du Service de Santée des Forces Armées en Nouvelle Calédonie, 98846 Nouméa Cedex, France; ^3^Antenne Vétérinaire de Paris, 1 Place Joffre, 75700 Paris SP 07, France; ^4^Unité de Recherche sur les Maladies Infectieuses et Tropicales Emergentes, I.H.U. Méditerranée Infection, 27 Boulevard Jean Moulin, 13385 Marseille Cedex 5, France; ^5^Laboratoire des Leptospires, VetAgro Sup, Campus Vétérinaire de Lyon, 1 Avenue Bourgelat, 69280 Marcy-l'Étoile, France

## Abstract

Leptospirosis is an important zoonotic disease in the world and a real public health concern for many years in New Caledonia. A cross-sectional survey was carried out on domestic and wild animals from New Caledonia in April 2009. Blood samples were collected from 30 cattle, 29 deers, (*Cervus timorensis russa*), 25 horses, 51 dogs, and 8 cats and were tested for 23 serovars of pathogenic *Leptospira* species by the microscopic agglutination test. From the total number of 143 samples, 84 (58.7%) were found to be positive towards one or several serovars of pathogenic leptospires. According to the species, the positive sera were obtained from 43% of 30 cattle, 72% of 29 Rusa deer, 80% of 25 horses, and 43% of 51 dogs, and fromall of the 8 cats tested. This study shows the broad dispersion and the high prevalence of the different serogroups of pathogenic *Leptospira* species tested, particularly among deer and horses. The disease is endemic in domestic animals and concerns all the species.

## 1. Introduction

Leptospirosis is presumed to be the most widespread zoonosis in the world [1, 2]. The disease is caused by spirochetes of the genus *Leptospira* and more than 260 serovars of pathogenic *Leptospira* species are now recognized [3]. Pathogenic strains are placed into 24 serogroups based on agglutinating antigens and into multiple genomospecies based on DNA studies [4, 5]. Leptospirosis has been identified as an emerging or a re-emerging infectious disease, particularly in tropical and subtropical regions, where environmental conditions favour the survival and transmission of leptospires [6, 7].

The global burden of disease is unknown because of the lake of data, but incidence estimates range from 0.1 to 1/100,000/year in temperate areas, to over 100/100,000/year during epidemics in the tropics. Estimated 300,000–500,000 severe cases occur each year, with case-fatality reports of up to 50% [8–12].

The disease constitutes a serious public health issue, particularly in areas which are known to be at high-risk for leptospirosis, as Pacific Islands [7]. Leptospirosis is known to be endemic in Pacific Islands and epidemics often occur during seasonal heavy rainfall and flooding and are associated with extreme weather events, notably under the influence of the El Nino Southern Oscillation [18], as exemplified by the outbreak in the Philippines in 2009, which occurred a month after Ondoy typhoon flooded Manila, causing 3382 cases and 249 deaths [13], or as supposed in 2008 [14, 15] for the epidemic human leptospirosis in New Caledonia, which caused 135 cases and 5 deaths. New Caledonia is located around 21°30′S 165°30′E/21.5°S 165.5°E, approximately, 1200 kilometres east of Australia and 1500 kilometres northwest of New Zealand, in the subregion of Melanesia, southwest Pacific. It comprises a main island (Grande Terre), the Loyalty Islands, and several smaller islands. The capital and largest city of the territory is Nouméa. 

In New Caledonia, 162 human cases were declared in 2009, with two deaths. The annual incidence is from 100 to 200 times higher in men in New Caledonia compared to those in the metropolitan France [16]. The incidence of infection is much higher in warm-climate countries than in temperate regions, due both to longer survival of leptospires in the environment in warm, humid conditions and to greater opportunities for human exposure [6]. Humans are usually infected through indirect exposure with a freshwater or humid environment contaminated with the urine of reservoir animals [17, 18]. Many of the serovars circulating in animal reservoirs have been shown to cause disease in humans. Infected animals are mostly asymptomatic, act as reservoir hosts to a particular serovar of leptospires, and shed the bacteria through their urine for prolonged periods of time. There is a wide range of animal hosts including wild or domestic animals, especially rodents and small marsupials, cattle, pigs, horses and dogs [3, 19]. 

The aim of the present study was to estimate the prevalence of leptospiral antibodies among domestic and wild animals in New Caledonia.

## 2. Materials and Methods

### 2.1. Animals

In April 2009, a cross-sectional survey was carried out in domestic and wild animal from New Caledonia. One hundred and forty three blood samples were collected from 30 cattle, 29 deers (*Cervus timorensis russa*), 25 horses, 51 dogs and 8 cats. *Cervus timorensis russa* was introduced to New Caledonia in 1862 from Indonesia (Java) and New Caledonia has one of the most important population of Rusa deer in the world [20]. The deer lives free in the savannah or in semi freedom, in big breeding farms on immense plot of land. The blood samples from the cattle and deers were collected by veterinarians in slaughterhouse of the office of marketing and refrigerated storing (OCEF) of Bourail (South Province).

Dog blood samples were collected in kennel, either in the municipal pound of Nouméa (South Province), or within Society for the Prevention of Cruelty to Animals (SPCA) (Dumbea, suburbs of Nouméa). Breed, sex, and age of dogs were varied and no data are available about the sanitary status or possible prophylaxis. Cat blood samples were collected in the municipal pound of Nouméa. Horse blood samples came partly from stables of Païta (suburbs of Nouméa), and the others from the Népoui police squad (North province). 

### 2.2. Laboratory Diagnostics

After centrifugation, sera were stored at −20°C before sending to France for microscopic agglutination tests (MAT) in the veterinary school of Lyon. The MAT is the serological reference test, particularly appropriate for carrying out epidemiological studies, since it can be applied to sera from any animal species, and because the range of antigens utilized can be expanded or decreased as required [6]. The MAT was performed using twenty-three serovars of pathogenic *Leptospira* species: Icterohaemorrhagiae, Copenhageni, Australis, Bratislava, Munchen, Autumnalis, Bim, Castellonis, Bataviae, Canicola, Hebdomadis, Panama, Mangus, Pomona, Pyrogenes, Sejroe, Saxkoebing, Hardjo, Wolffi, Tarassovi, Cynopteri, Vanderhoedoni, and Grippotyphosa. The choice of the serovars used for each serogroup was based upon the experience of the laboratory. According to observations recorded in the French *Leptospira* laboratory in Nantes and later at Lyon, titers as low as 1 : 40, 1 : 100, and 1 : 200 were considered as positive agglutination reactions respectively for carnivores, ruminants, and horses. The end-point is the highest dilution of serum in which 50% agglutination occurs. 

### 2.3. Statistical Analysis

The results were analysed by 2 × *K* contingency tables of exposure variables. The outcome variable was positivity to leptospiral antibodies (MAT) and the independent variables were: age, sex, and species. Odds ratios (OR), 95% confidence interva (CI) and *P* values were calculated separately for each variable using the Epi info (version 5.01, CDC Atlanta, USA). The Chi-square, or the Fisher exact test if appropriate, was used to evaluate associations (*α* = 5%). Differences were considered statistically significant when *P* ≤ 0.05. Cattle, horse, deer and dogs were classified into age groups, which differed according to species. However the low numbers examined and positive animals in some species determined the low number of age groups formed to allow for statistical analysis. Accordingly, deer, horses and cattle were all classified into two age groups, young and adults, which were defined as follows: deer, ≤2 year old, *n* = 19, and >2 year old, *n* = 10; horses, ≤6 year old, *n* = 11, and >6 year old, *n* = 14; cattle, <4 year old, *n* = 17, and ≥4 year old, *n* = 13. Dogs were classified into three age groups: young (≤2 years of age; *n* = 15), adult (between 2–8 year old; *n* = 24) and senior (≥8 year old; *n* = 12). Cats were not classified into age groups due to a lack of data.

## 3. Results

**Figure 1:**
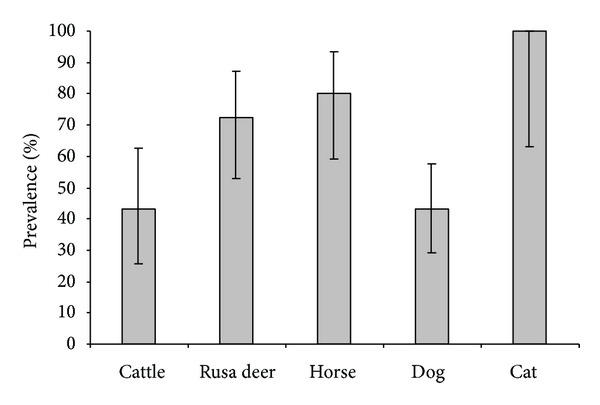
Percentage of positive samples for leptospirosis (MAT), according to species. Error bars indicate 95% confidence intervals.

From the total number of 143 samples, 84 (58.7%) were found to be positive when a cut off of 1 : 40, 1 : 100, and 1 : 200 or higher was respectively applied for carnivores, ruminants and horses. Leptospiral prevalence according to respective species was shown in Figure 1. No statistically significant difference of prevalence due respectively to sex and to age was observed in cattle (*P* = 0.5; *P* = 0.1), horses (*P* = 0.3; *P* = 0.5), deer (*P* = 0.7; *P* = 0.5), and dogs (*P* = 0.2; 0.7). Except cats, there is no statistically significant difference between different species (*P* > 0.05).

Positivity can be observed for one or more leptospiral antigens, so we have recorded 338 positive serological reactions with different serovars for the 84 positive samples. The results of positive MAT according to leptospiral serovars in different animal species are shown in Table 1. 

**Figure 2:**
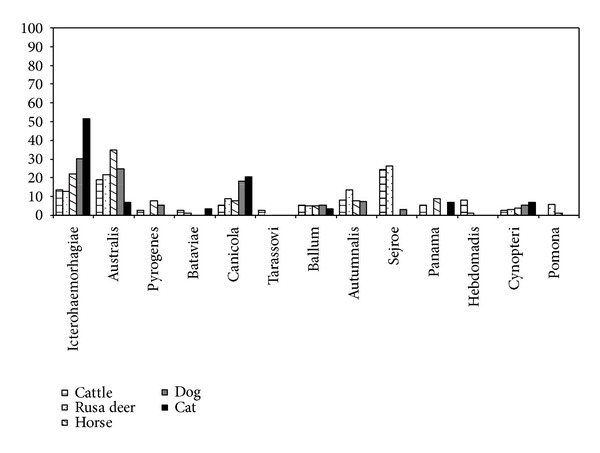
Distribution of different serogroups of pathogenic *Leptospira* species in ruminants, horse, and carnivores.

**Figure 3:**
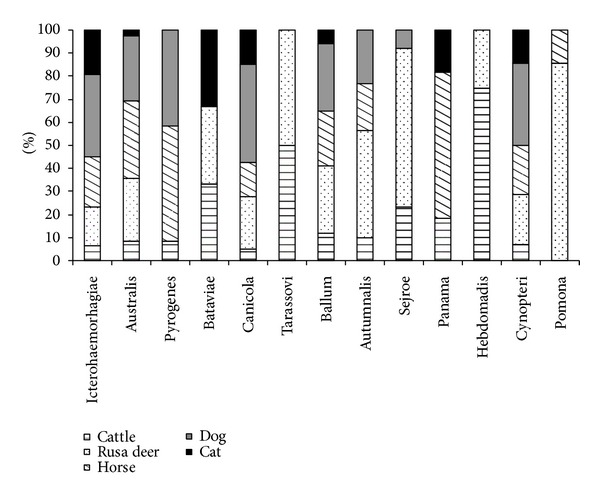
Distribution of animal hosts by serogroup.

The distribution of different serogroups among the seropositive animals, according to species is presented in Figure 2.  Figure 3 shows distribution of animal hosts by serovars. 

Each histogram stands for the percent contribution of each host on the total positive hosts for the whole given serogroup. All serogroups were considered on a basis of a similar scale.

The obtained results in Figure 2 indicate serogroup Sejroe as the most frequent serogroup (24%) in cattle. The most frequent serogroup in deer, horse, dog and cat are respectively Sejroe (26%), Australis (35%), and Icterohaemorhagiae (30% and 52%).

Although the low number of individuals per group did not allow for the application of statistical tests of comparison, it is possible, in some extent, to indicate from the Figure 3 that the Icterohaemorrhagiae, Australis, Canicola, Ballum and Cynopteri serogroups circulate in all the hosts investigated; within the Icterohaemorrhagiae positive hosts, dogs were most frequently infected (36%) whereas Pomona, Sejroe and Autumnalis where of higher prevalence in deers (86%, 69%, and 47%) in contrast to Pyrogenes which was mainly restricted to horses (50%) and dogs (42%) as were the serogroups Hebdomadis and Tarrassovi exclusive to ruminants. Finally horse (64%) was the major host for Panama serogroup. Details by animal species are given below.

### 3.1. Cattle

Of the 30 cattle tested, 43% were found to be seropositive (Figure 1). Antibody titers ranged from 200 to 3200 (50% ≥ 800). Serovars Autumnalis and Mangus showed the highest antibodies titers (maximum dilution = 1 : 3200). Of the 13 positive sera, 5 reacted to one serogroup only, 4 showed cross reactions in which one serogroup predominated, and 4 reacted with two or more serogroups at the same degree. When one serogroup predominated, Ballum was the most frequently recorded (33%), followed by Sejroe (22%). 

### 3.2. Rusa Deer

Of the 29 Rusa deer tested, 72% were found to be seropositive (Figure 1). Antibody titers ranged from 100 to 6400 (51% ≥ 800). Serovars Ballum, Sejroe, Autumnalis, and Australis showed the highest antibodies titers (maximum dilution = 1 : 6400). Of the 21 positive sera, 7 reacted to one serogroup only, 6 showed cross reactions in which one serogroup predominated, and 8 reacted with two or more serogroups at the same degree. When one serogroup predominated, Sejroe was the most frequently observed (46%), followed by Autumnalis (23%) and Ballum (15%). 

### 3.3. Horse

Of the 25 horses tested, 80% were found to be seropositive (Figure 1). Antibody titers ranged from 200 to 6400 (21% ≥ 800). The highest antibody titer was recorded for serovar Icterohaemorrhagiae (maximum dilution = 1 : 6400). Of the 20 positive sera, 12 showed cross reactions in which one serogroup predominated, and 8 reacted with two or more serogroups at the same degree. When one serogroup predominated, Icterohaemorrhagiae was the most frequently recorded (50%), followed by Australis (42%). 

### 3.4. Dog

Of the 51 stray dogs tested, 43% were found to be seropositive (Figure 1). Among these 22 positive dogs, 13 had a compatible profile with vaccine profile, 6 had an infection (>1 : 320), among which 4 towards the serovar Copenhageni (1 : 2560, max dilution), 1 towards the serovar Pyrogenes (1 : 2560), and 1 towards the serovar Sejroe (1 : 320). For 3 positive dogs it was not possible to individualize a dominant serogroup. No data was available about the sanitary status or possible vaccination of the stray dogs. No symptoms of disease were identified in any of the dogs included in the study.

### 3.5. Cat

All of the 8 cats tested were positive (Figure 1). Antibody titers ranged from 40 to 1280 (24% ≥ 320). Serovars Canicola and Ballum showed the highest antibodies titers (maximum dilution = 1 : 1280). Of the positive sera, 2 reacted to one serogroup only, 2 showed cross reactions in which one serogroup predominated, and 4 reacted with two or more serogroups at the same degree. When one serogroup predominated, Icterohaemorrhagiae was the most frequently recorded (75%), followed by Canicola (15%).

## 4. Discussion

In our study, 58.7% of animals are positive towards one or several serovars of pathogenic leptospires, without evidence of clinical leptospirosis. These results confirm that the majority of animals which were positive have survived to leptospiral infections or are asymptomatic. The presence of antibodies indicates that animals were exposed to the pathogen.

However, the cut-off points selected (low in epidemiological investigation), and the absence of kinetic serology do not allow in most cases to conclude in a current active infection. 

Clinical signs are quite variable and most cases are probably inapparent and associated with host-adapted serovars such as Canicola in dogs, Bratislava in horses, Hardjo in cattle [19, 21]. 

Vaccination is also involved in interpreting the results. It is relatively little used in cattle in New Caledonia and the vaccine protects against three strains: Hardjo, Copenhageni and Pomona. In dogs, the vaccine used in New Caledonia protects against Icterohaemorrhagiae and Canicola and does not prevent carriage or excretion. 

The high prevalence and the large number of serovars reacting positively in our study are close to those observed frequently in serological surveys conducted in tropical or equatorial areas. The high biodiversity of the serovars is generally related to the wide range of mammalian reservoir [10]. In New Caledonia, rodents are a major reservoir of leptospirosis. The three species of rats (black, Polynesian and common rats), and the mice were introduced during the various episodes of settlement of New Caledonia and contribute to maintain and to transmit serogroups Icterohaemorrhagiae and Ballum respectively [14, 15]. 

Except to the cat, the rate of leptospirosis infection does not vary significantly according to the species (at the 5% risk) in our study. However, the sample size limits the scope of this result, and a study in the Fiji Islands revealed a prevalence of 0% in cats (0/3) [22]. The prevalence rate observed in dogs in our study (43%, 22/51) is close to that observed in dogs in the Fiji Islands (56%, 19/34). Dogs are not usually considered as a reservoir for *Leptospira*, except for Canicola [6, 19]. 

In our study, we observe four possible infections by Pyrogenes strains. This serogroup is rarely encountered in serological surveys conducted in dogs, but is one of the most common serogroups identified in humans over the period 2005–2009 in New Caledonia, with Australis and Icterohaemorrhagiae serogroups [16]. 

The prevalence rate observed in horses in our study (72%; 18/25) is close to that previously obtained in the Fiji Islands 83% 19/23). The prevalence rate observed for cattle in our study (43%; 13/30) is significantly lower (*P* > 0.01) than that published for Fiji Islands cattle (70%; 128/183).

Compared to results of surveys conducted between 2005 and 2009 in New Caledonia the serogroup Pomona is not particularly associated to cattle, horses and neither deer in our study. In contrast, we were able to associate the serogroup Australis to these species, while it was not the case in the previous studies [16].

## 5. Conclusion

This study shows the broad dispersion and the high prevalence different leptospiral serogroups, particularly among deer and horses. The disease is endemic in domestic animals and concerns all the species. The diversity of serovars detected is indicative of the possible existence of a variety of animal reservoirs. 

Leptospirosis is an important zoonotic disease in New Caledonia and is a real public health problem in both human and animal. The incidence from 100 to 200 times higher in men in New Caledonia compared to the metropolitan France is associated with the tropical climate and the practice of breeding. The main serogroups identified in human cases are also significantly found in animals. This is the case of Icterohaemorrhagiae (dogs), Australis (cattle, horses, deer), and Pyrogenes (dogs). Additional studies are required to better understand the complexity within the epidemiology of leptospirosis in New Caledonia and to identify the reservoirs for the different serogroups. 

## Figures and Tables

**Table 1:** Results of positive MAT according to pathogenic leptospiral serovars in different animal species (only reactive serovars are listed).

Serogroup	Serovar	Cattle	Deer	Horse	Dog	Cat	Total
Icterohaemorrhagiae	Icterohaemorrhagiae	1	2	1	12	7	23
	Copenhageni	4	11	16	16	8	55
Australis	Munchen	3	10	9	5	2	29
	Australis	2	6	8	6	—	22
	Bratislava	2	6	10	12	—	30
Pyrogenes	Pyrogenes	1	—	6	5	—	12
Bataviae	Bataviae	1	1	—	—	1	3
Canicola	Canicola	2	9	6	17	6	40
Tarassovi	Tarassovi	1	1	—	—	—	2
Ballum	Castellonis	2	5	4	5	1	17
Autumnalis	Autumnalis	1	5	—	—	—	6
	Bim	2	9	6	7	—	24
Sejroe	Sejroe	2	6	—	3	—	11
	Wolffi	3	11	—	—	—	14
	Hardjo	4	10	—	—	—	14
Panama	Mangus	2	—	4	—	1	7
	Panama	—	—	3	—	1	4
Hebdomadis	Hebdomadis	3	1	—	—	—	4
Cynopteri	Cynopteri	1	3	3	5	2	14
Pomona	Pomona	—	6	1	—	—	7

Total		37	102	77	93	29	338
